# Added Value of a Previous Exam Comparison in the Final Breast Imaging and Reporting Data System (BI-RADS®) Assessment in Screening Mammography

**DOI:** 10.7759/cureus.72616

**Published:** 2024-10-29

**Authors:** Eduardo De Faria Castro Fleury, Veronica J Ayres

**Affiliations:** 1 Radiology, MD duFLE Diagnósticos, São Paulo, BRA; 2 Mastology, Faculdade de Medicina do ABC, São Paulo, BRA

**Keywords:** breast cancer biology, breast cancer pathology, breast imaging reporting and data system (bi-rads), breast screening, mammography, mammography usage

## Abstract

Purpose: This study aims to determine the impact of prior examination comparison in the final Breast Imaging and Reporting Data System (BI-RADS®) assessment in screening mammography. We compared the incidence of BI-RADS® categories 0, 1, 2, 3, 4, and 5 before and after comparing to the previous exam, with a focus on category 0. We also compared the radiologists' final classification according to their clinical practice experience.

Materials and methods: This is a prospective observational study conducted from August 2020 to February 2021. Two radiologists independently interpreted 3,896 consecutive mammograms. The final BI-RADS® category was given for the mammography blinded to the previous examinations and after comparing the previous examination. We also compared the radiologists' final classification according to their clinical practice experience.

Results: A total of 3,656 patients referred for breast mammography were evaluated, and 3,531 screening mammograms were included. The final BI-RADS® classification for mammograms blinded to previous examinations was as follows: 389 (11.02%), 3,017 (85.44%), 35 (0.99%), 87 (2.46%), and 3 (0.08%) for categories 0, 1, 2, 3, 4, and 5, respectively. After comparing the previous examinations, the results were as follows: 103 (2.92%), 3,311 (93.77%), 39 (1.10%), 75 (2.12%), and 3 (0.08%) for categories 0, 1, 2, 3, 4, and 5, respectively. Reader 1 read 1,439 mammograms, while reader 2 read 2,093 mammograms. When comparing the final category of each reader independently, reader 1 classified the mammograms as 91 (7.10%), 1,181 (92.19%), two (0.16%), and seven (0.55%) for blinded mammograms and 0, 1,261 (98.44%), six (0.47%), and 14 (1.09%) for mammograms after prior examination comparison for categories 0, 1 and 2, 3 and 4, respectively. Reader 2 classified the mammograms as 194 (9.27%), 1,181 (87.52%), 27 (1.29%), and 40 (1.91%) for blinded mammograms and one (0.05%), 2,044 (97.71%), 26 (1.24%), and 21 (1.00%) for mammograms after previous exams comparison for categories 0, 1 and 2, 3, and 4, respectively.

Conclusion: Comparing previous mammograms of participants in breast cancer screening programs may reduce the number of BI-RADS® category 0 final classifications by 73.8% and the number of positive findings in the final classification, especially for the less experienced readers.

## Introduction

Breast cancer is the main cancer diagnosed in women in Brazil, and mammography is the primary test for screening and early detection of this tumor [1]. Breast cancer screening with mammography is accepted and recognized globally and has been established as a public health policy program in most countries [2].

Studies show that mammography screening reduces breast cancer mortality by 20% to 40% and enables early diagnosis at subclinical stages [3,4]. Early diagnosis allows for more conservative, cost-effective, and efficient treatments. The cost of mammographic screening is lower than that of treating advanced cancer, which justifies the application of this test as a public health promotion program [5].

Screening programs must be adapted to the public health realities of each country and can be organized or opportunistic. Organized programs should cover the entire population eligible for screening for the target disease, where the patient is invited to voluntarily join the program. In opportunistic screening, the patient is referred for screening by a qualified professional. Breast cancer screening comprises a diagnostic tripod in which, in addition to mammography, clinical examination data and cytopathological analysis of positive findings are valued in the diagnosis and management of lesions [6].

As it is a screening program, it must be systematic and continuous, with three mammography types to be evaluated: (i) first examination, (ii) screening examination, and (iii) diagnostic examination. The mammogram from the patient's (i) first examination will be the reference for all screening when it is negative. If it is positive, the patient will need further assessment with ultrasound and a possible biopsy. Lesions with benign characteristics can be monitored for an interval of six months, one year, and two years to assess the stability of the lesion. The second standard, (ii) screening, is an annual or biannual control using previous mammograms as a reference. Screening mammograms correspond to four mammogram incidences, comprised of bilateral mid-lateral oblique and craniocaudal views. Finally, (iii) diagnostic mammograms consist of studies of patients with clinically active alterations (lesions between screening mammograms), positive findings in the first test, or new findings in the screening tests compared to previous tests. In diagnostic mammograms, complementary views in mediolateral incidence, focused compression, or magnification can be added to the four standard views [7].

Mammography findings are classified according to the Breast Imaging and Reporting Data System (BI-RADS®) lexicon, devised by the American College of Radiology (ACR), currently in its fifth edition. Mammography findings are assessed by two criteria: the first consisting of the morphological description of the lesion and the second the temporal analysis of the lesion's evolution, which evaluates the stability of dimensions and morphological changes. The final BI-RADS® classification has seven categories where (i) category 0 corresponds to incomplete findings and requires further evaluation, (ii) categories 1 and 2 are negative categories for suspicious lesions, (iii) category 3 are probably benign lesions that need to be followed-up in short intervals, categories (iv) 4 and 5 are suspicious for malignancy and need histopathologic correlation, and category (v) 6 are lesions with a positive diagnosis of breast cancer. Categories 0, 3, 4, and 5 are positive [1,7].

False-positive and false-negative results are the weak points of any screening program, which is no different from mammography screening. One way to reduce false-positive and false-negative results is to compare the current findings with previous examinations. According to the BI-RADS® lexicon, comparison to previous examinations, if deemed appropriate by the interpreting physician, is recommended: “Comparison to previous examinations may assume importance if the findings of concern require an evaluation of change or stability. Comparison is not important when a finding has unequivocally benign features. Comparison may be irrelevant when the finding is inherently suspicious for malignancy” [8].

The mammographic screening program is a secondary prevention of breast cancer, i.e., it doesn't prevent the disease but allows for early diagnosis and thus reduces the impact of the disease's morbidity and mortality [9]. Basically, breast cancers are classified by histology and immunohistochemical profile. The immunohistochemical profile is related to the aggressiveness of the disease, where estrogen and progesterone hormone receptors are assessed, as well as human epidermal growth factor receptor 2 (HER-2) receptors and cell duplication activity (KI67). Tumors can be divided into luminal A and B (less aggressive), HER-2, and triple-negative (more aggressive). Luminal cancers have a longer cell duplication time and are more likely to be detected in screening programs, while triple-negative cancers, because they are more aggressive, are less likely to be detected in screening programs [10,11].

Patients participating in a breast cancer screening program with positive mammography findings and no previous tests available for comparison should be considered as institutional category 0 at the referral service. According to the BI-RADS® lexicon, the service has a period of three weeks to request previous exams for comparison purposes and, when available, compare them with the current exam and define the final classification according to the lexicon. If the previous exams are unavailable, the mammogram should be classified as category 0, and the investigation should continue with complementary scans or ultrasonography [8].

This study aimed to assess the impact of comparing previous mammograms on the final classification proposed by the BI-RADS® lexicon in patients participating in the breast cancer screening program at an institution dedicated to diagnosing and treating breast cancer in São Paulo, Brazil. We also compared the radiologists' final classification according to their clinical practice experience.

## Materials and methods

This prospective, observational, single-center study, approved by the institutional ethics committee and with an informed consent form signed by the patients, was registered on the Brazil Platform under "Certificado de Apresentação de Apreciação Ética" (CAAE): 24043619.3.0000.0072.

Patients referred for breast cancer screening mammography to the Diagnostic Imaging service were evaluated consecutively between August 2020 and February 2021. Two researchers, with five and 20 years of experience, independently read the mammograms using a standardized and auditable report form to describe the findings and the final classification, as proposed by the current edition of the BI-RADS® lexicon.

**Figure 1:**
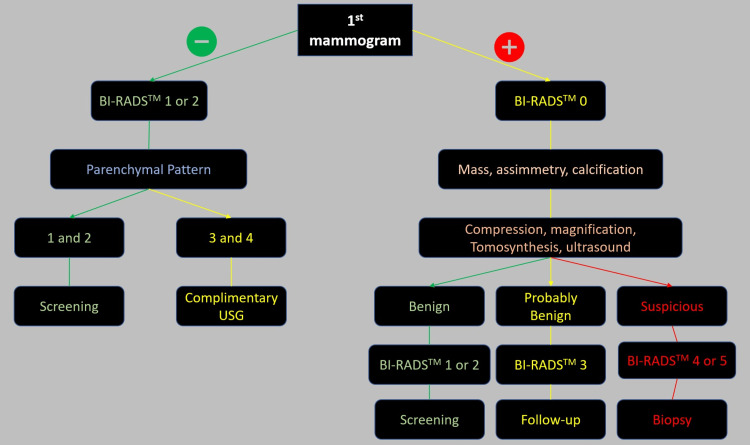
Workflow for the first mammography We have only BI-RADS® 0, 1, and 2 at the first assessment. After complimentary studies, probably benign findings should be considered for benign features without evolutionary control. BI-RADS®: Breast Imaging and Reporting Data System, USG: ultrasonography

**Figure 2:**
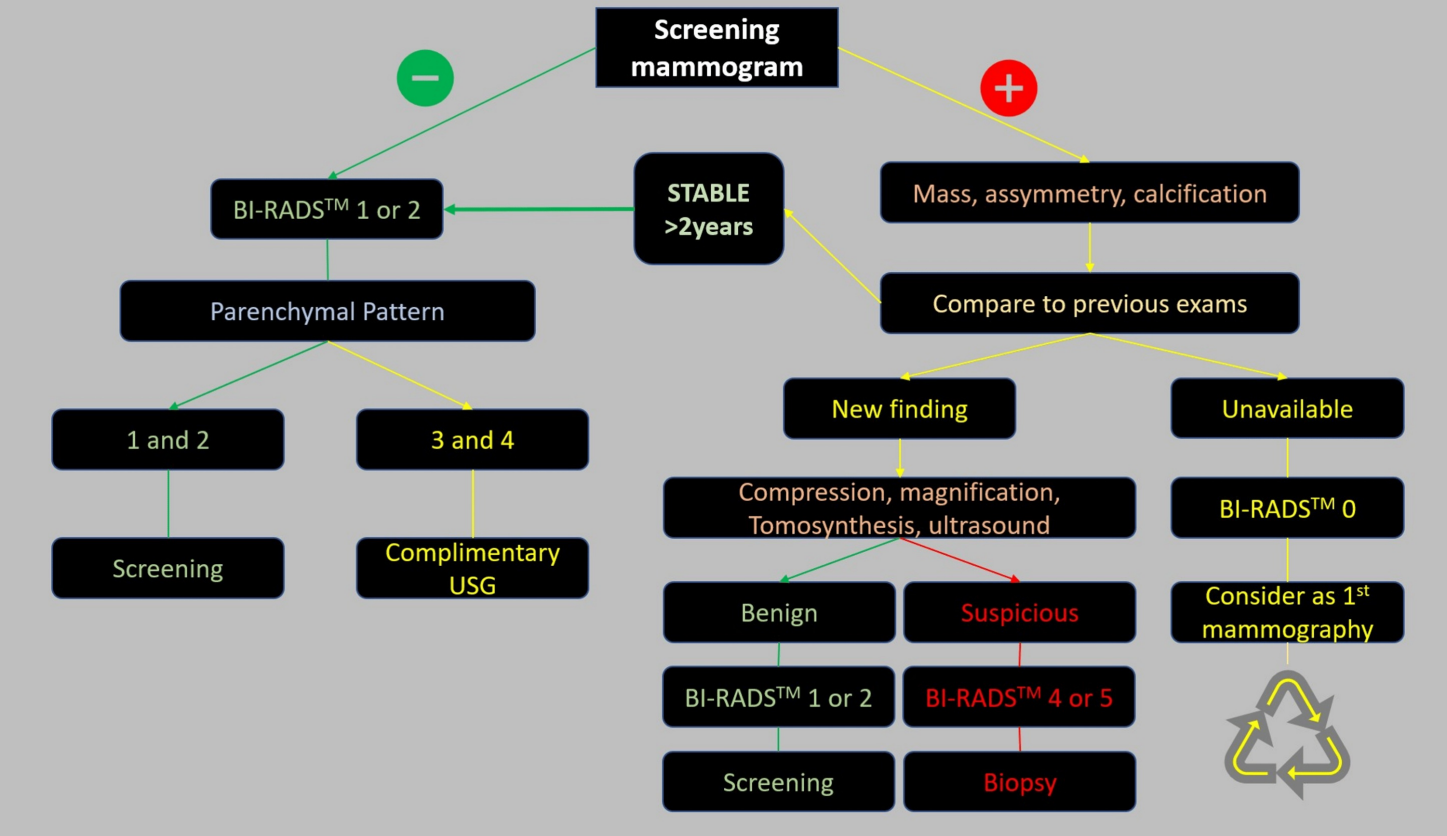
Workflow for screening mammogram We should not have BI-RADS® 3 classification. BI-RADS® 0, 1, and 2 at first assessment. After comparison to previous studies, benign or malignant assessment should be reported. When the previous exams are unavailable, a vicious circle should be formed. BI-RADS®: Breast Imaging and Reporting Data System, USG: ultrasonography

The flow of patients in the service varies according to the type of mammogram performed, with specific flows for the first exam (Figure 1) and screening exams (Figure 2). Mammography was performed using the Fujifilm Amuled full-field digital mammography system (Fujifilm, Tokyo, Japan). Standard craniocaudal and mid-lateral oblique views were routinely obtained.

The mammograms of patients who met the inclusion criteria were evaluated in two rounds (Table 1).

**Table 1:** Study design BI-RADS®: Breast Imaging and Reporting Data System

	Exam	Indication	First round	Second round
Exclusion		Diagnostic, 1st read		
Inclusion	Mammogram	Screening	Reader 1, reader 2	Reader 1, reader 2
Description	4 incidences, standard protocol	Inclusion criteria: >40 years, asymptomatic	BI-RADS® classification: blinded to previous exams, final assessment	BI-RADS® classification: comparison to previous exams, final assessment

In the first round, the researchers evaluated the four standard mammogram images. Based on the findings, they issued a final report, ignoring the results of previous examinations, as was done when interpreting the first mammogram in clinical practice.

In the second round, immediately after the first evaluation, the researchers added the information from the previous exams to the report and issued the final report. At least one previous negative mammogram with an interval of less than two years was considered a previous exam when it was available on the picture archiving and communication system.

The final classifications of each round were compared, using as a reference an article published in 2005 that audited a screening program (Table 2) [12]. The final classifications of blind mammograms and mammograms with previous examinations were compared. The distributions of the final classifications for each reader were also compared.

**Table 2:** Results of the audited screening program A multicenter study of mammography. Audit of a screening program. The authors report 8.51% of BI-RADS® 0 and 0.16% of BI-RADS® 3 final assessment. BI-RADS®: Breast Imaging and Reporting Data System

BI-RADS®	N	%
0	3,623	8.51
1 and 2	38,829	91.25
3	68	0.16
4	23	0.05
5	9	0.02

Significance values were considered when p<0.05. MedCalc for Windows software version 19.4 (MedCalc Software, Ostend, Belgium) was used to statistically analyze the results.

## Results

From August 2020 to February 2021, 3,656 mammograms were evaluated. One hundred twenty-five mammograms were excluded because they did not meet the inclusion criteria. A total of 3,531 patients were included in the study, of whom 378 had previous examinations. Reader 1, with 20 years of experience, read 1,439 mammograms, while reader 2, with 5 years of experience, read 2,093 mammograms.

Table 3 shows the distribution of the BI-RADS® categories for all the patients, comparing the classifications of the first round (blinded) with the added classification of the association with the previous exams and the final classification. Table 4 shows the distribution of results for the patients included in the screening program.

**Table 3:** Distributions of BI-RADS® final assessment for all mammograms, blinded to previous exams, the results of the previous exams, and the final assessment BI-RADS®: Breast Imaging and Reporting Data System

BI-RADS®	Blinded		With previous		Final	
0	422	11.54%	0	0.00%	114	3.12%
1 and 2	3072	84.03%	334	79.71%	3389	92.70%
3	39	1.07%	35	8.35%	42	1.15%
4	107	2.93%	44	10.50%	91	2.49%
5	10	0.27%	0	0.00%	10	0.27%
6	6	0.16%	6	1.43%	10	0.27%
	3656		419		3656	

**Table 4:** Distributions of BI-RADS® final assessment for screening mammograms, blinded to previous exams, the results of the previous exams, and the final assessment BI-RADS®: Breast Imaging and Reporting Data System

BI-RADS®	Blinded		With previous		Final	
0	389	11.02%	0	0.00%	103	2.92%
1 and 2	3017	85.44%	310	82.01%	3311	93.77%
3	35	0.99%	33	8.73%	39	1.10%
4	87	2.46%	35	9.26%	75	2.12%
5	3	0.08%	0	0.00%	3	0.08%
6	0	0.00%	0	0.00%	0	0.00%
	3531		378		3531	

**Figure 3:**
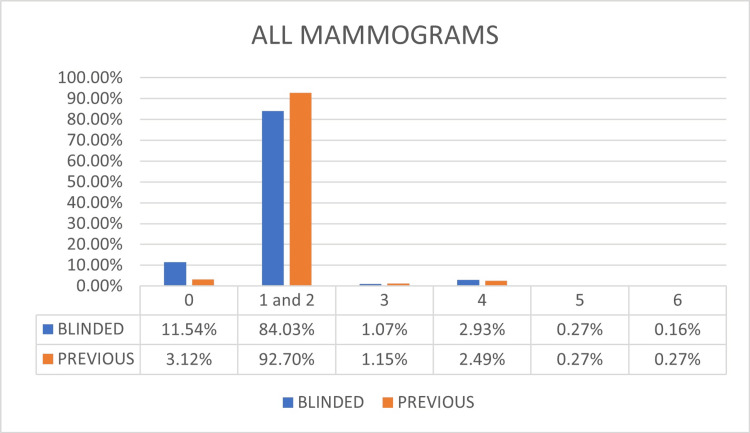
Results of the final category assessment for all mammograms comparing the blinded reads with the mammograms with previous exams The BI-RADS® 0 final assessment decreased from 11.54% to 3.12% compared to previous exams. Consequently, the BI-RADS® 2 increased from 84.03% to 92.70%. There was also a decrease in the BI-RADS® 4 final assessment without statistical significance. BI-RADS®: Breast Imaging and Reporting Data System

Figure 3 shows the comparative graph of the distribution of each category for all the patients referred to the service. It can be seen that the BI-RADS® 0 final assessment decreased from 11.54% to 3.12% when added to the previous exam comparison. Consequently, the BI-RADS® 2 increased from 84.03% to 92.70%. There was also a decrease in the BI-RADS® 4 final assessment.

**Figure 4:**
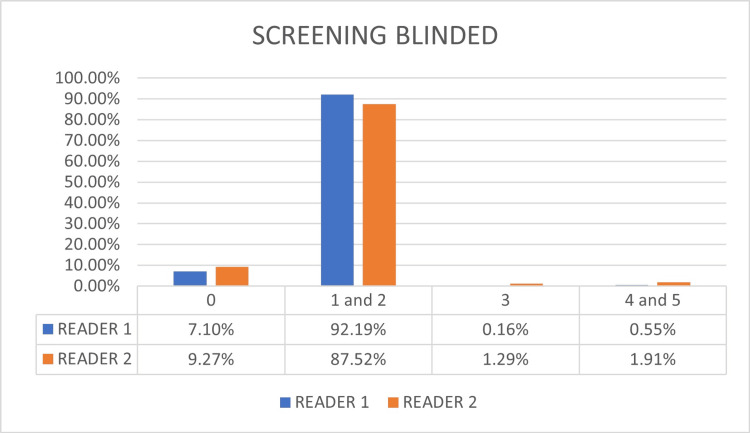
Results of the final category assessment for screening blinded to previous exams comparing the readers Audit results for screening mammograms blinded to previous exams. The less experienced reader tends to have more BI-RADS® 0 final assessment (9.27% vs. 7.10%). There are also statistical differences in the BI-RADS® 3 assessment (1.29% vs. 0.16%). BI-RADS®: Breast Imaging and Reporting Data System

Reader 1 used previous exams for comparison in 7.2% of cases, while reader 2 compared in 12.0%. Figure 4 shows the comparative graph of the distributions of each category for each investigator without the assessment of the previous exam in patients where the comparison was performed. It can be seen that the less experienced reader tends to have more BI-RADS® 0 final assessment (9.27% vs. 7.10%) without previous exams. There are also statistical differences in the BI-RADS® 3 category (1.29% vs. 0.16%) and BI-RADS® 4 category (1.91% vs. 0.55%).

**Figure 5:**
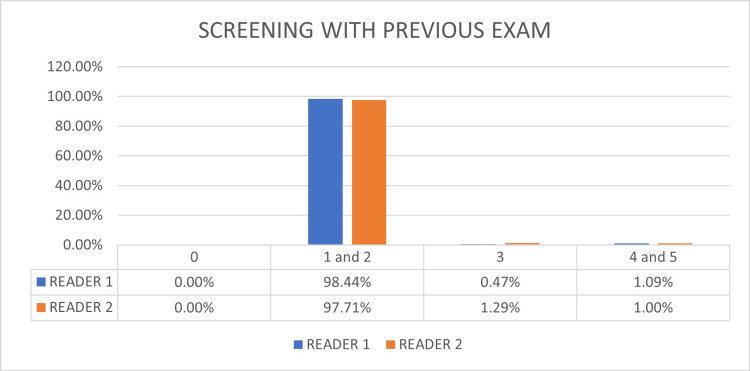
Results of the final category assessment for screening mammograms with previous exams comparing the readers When comparing the impact of previous studies comparison, there is an improvement in the reader 2 final assessment. The previous studies' comparison leveled reader 2 to the same results as reader 1.

Figure 5 shows the comparative graph of the distributions of each category for the final result for patients with previous examinations according to the final classification of each investigator in patients where the comparison was performed. There is an improvement in reader 2's final assessment when comparing the impact of prior studies. The comparison between the previous studies leveled reader 2 to the same results as reader 1. However, there was still a statistically significant difference in category 3 between the researchers (1.29 vs. 0.47).

Table 5 shows the significance level of the comparison between the readers for each final category in the blinded exams and the comparison with previous exams.

**Table 5:** Significance values for the final classifications of mammograms for Readers 1 and 2 assuming equal variances with a significance level of p<0.05

BI-RADS® category	p-value
Blinded to previous exams	
All categories	0.8212
0	0.0236
1 and 2	<0.0001
3	<0.0001
4	0.0002
With previous exams	
All categories	0.4528
1 and 2	0.1487
3	0.0121
4	0.8044

The study's results show an overall reduction in category 0 in patients in the breast cancer screening program from 11.02% to 2.92%, a reduction of 73.50%, and a reduction in category 4 from 2.46% to 2.12%, a reduction of 13.82%.

## Discussion

Mammography is the primary imaging method used for breast cancer screening and diagnosis. Mammographic screening is the subject of numerous studies discussing its efficiency in public health programs. There is now recognition of the method's contribution to early diagnosis of the disease and its impact on patient survival. However, there is still criticism of the cost-effectiveness of breast screening programs in relation to public health (Table 6) [6,13,14].

**Table 6:** Criteria for a screening program and status of mammography * Criteria for a population screening program from the UK National Screening Committee updated September 29, 2022 [15].

Criteria for screening program*	Screening mammography
1. Health problem: target disease	Standard 4 incidences
2. Natural history: well known	Available and reproducible
3. Benefit: early diagnosis > natural manifestation	Do not need extra hardware, software, or storage
4. Test: available, acceptable, and reliable	Cheap
5. Cost: reasonable, compatible with budget	Analalogic or digital
6. Frequency: contínuos and systematic	Fullfill all criteria

The ideal breast screening program should be organized, with the data available in a single central database to search each patient's history. Patients should be invited during the established period to undergo the tests, and previous images should be available for comparison. However, this model is difficult to implement in countries where healthcare is decentralized and requires a high level of investment and organization to centralize and make the information accessible. As a result, screening is routinely carried out inadequately in many countries, especially due to the lack of comparison with previous examinations for issuing the final report. Non-comparison with previous tests invalidates the concept of systematic and continuous analysis of results, making screening inefficient [16-18].

**Figure 6:**
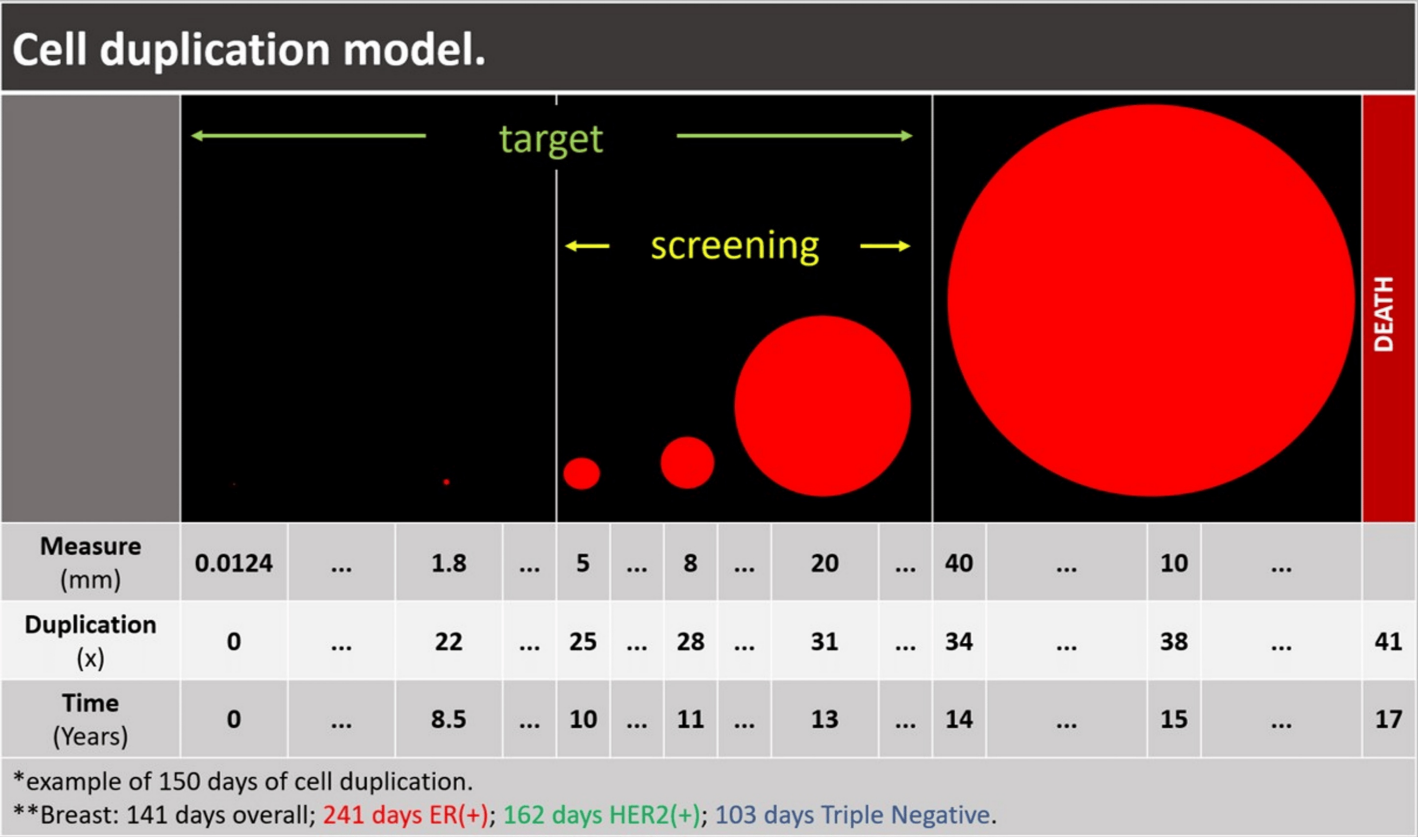
Cell duplication model and screening According to tumor biology, screening is more effective for luminal A and B tumors. Familiarity with tumor biology is essential for understanding the screening targets. The screening is due to the 25-31 duplications round.

Based on a reference article on cell duplication in lung cancer, we simulated the natural history of breast cancer from the pre-clinical periods and the role of screening in early detection [19]. Considering the cell duplication time according to the histochemical profile of breast cancer, it can be seen that the lesion becomes visible on the image at 0.5 cm, equivalent to 25 cell duplications and 10 years of evolution for tumors with a cell duplication time of 150 days. To reach 2.0 cm in diameter, 31 duplications and 13 years of evolution are required. This interval where imaging methods detect the lesion corresponds to six cell duplication intervals and three years of evolution, which justifies the annual or biennial screening interval without interfering with the prognosis of the disease. Following these criteria, screening becomes more efficient for luminal tumors, which are more indolent and metastasize later (Figure 6).

Many indicators can be adopted to access the quality control of breast screening facilities. According to Tomazelli et al., one of the criteria for assessing the quality of screening exams would be result distribution by acceptable parameters, as per the American College of Radiology, BI-RADS® category 0 under 12% (desirable = 5-12%) and BI-RADS® category 1 or 2 under 75%. The authors also discuss the recommendation of the American Medicare health system that the proportion of BI-RADS® 3 screening mammograms should be close to zero. The results reported by the authors in their article evaluating breast cancer screening indicators in the female population using the 2018-2019 National Health System in Brazil are similar to the results obtained in our study. However, the authors discuss the prevalence of category 3 in some published articles in Brazil. For the authors, "it is possible that studies using data from examinations without delimiting whether they relate to the same women and without adopting data quality assessment criteria may have artificially increased the proportion of BI-RAD® 3 results, as may have studies that addressed older women" [1].

Category 3 is probably benign. Lesions with morphological characteristics of benignity such as (i) an oval, circumscribed, medium-density mass, (ii) focal asymmetry, and (iii) a group of round calcifications should fall into this category when present on first examination. As the characteristics are benign but impossible to assess stability, further examinations are suggested at six-month, one-year, and two-year follow-ups to monitor dimensions or assess morphological changes. It is, therefore, understood that category 3 should be restricted to the first examination of patients [8]. In Brazil, the difficulty in accessing previous exams may be one of the reasons for the high number of exams classified in category 3 outside the standards adopted in the literature, probably the result of misconduct [1].

Many studies still discuss the importance of using complementary methods to mammography to reduce the number of incomplete results classified in category 0 and discuss using more modern methods such as tomosynthesis to achieve this goal. Studies show that tomosynthesis reduces the number of mammography recalls by 40% [15-17]. In the current study, our results show that the evaluation of previous studies reduces conventional mammography recalls by 73.8%. Our results also indicate that the prevalence of positive lesions classified in categories 3 and 4 may relate more to the examiner's experience than the method used. In our study, the less experienced examiner had more lesions classified in categories 3 and 4. Therefore, routine audits and continuing education programs aimed at the altered indicators can be used to improve the diagnostic accuracy of the services.

The graphs presented in this manuscript also show that after comparison with previous examinations, the more experienced examiner increased the number of results in categories 3 and 4. This is due to the redistribution of cases in category 0. For reader 2, there was an increment in category 2 and a reduction in lesions classified in categories 3 and 4. Analysis of the graphs shows that comparison with previous examinations brought the results between the examiners closer together in categories 2 and 4. The results reflect the tendency of the less experienced examiner to note positive results when data from previous exams is unavailable, corroborating the hypothesis that positive results when reading mammograms are directly related to the investigator's experience.

Our study has some limitations. Firstly, it was a single-center study with only two examiners. Multicenter studies with more examiners are needed to validate our findings. As the study aimed to evaluate the classification distribution according to the BI-RADS® lexicon before and after comparison with previous examinations, the results were not compared with the anatomopathological findings of the lesions classified as positive. In the future, we could include biopsy data and the results of long-term follow-up of these patients.

## Conclusions

Comparing previous mammograms of participants in breast cancer screening programs may reduce the number of BI-RADS® category 0 final classifications by 73.8% and the number of positive findings in the final classification, especially for the less experienced readers. Audits for the reader's performance allow a continued educational program to improve the quality of breast screening centers.

Mammography still has unique characteristics that validate it as the gold-standard imaging test for the breast cancer screening program, such as simplicity, availability, acceptance, cost, and reproducibility.

## References

[1] Tomazelli J, Dias MB, Ribeiro CM, Assis M, Pla MA, Canella EO, Migowski A (2023). Evaluation of breast cancer screening indicators in the female population using the National Health System, Brazil, 2018-2019: a descriptive study. Epidemiol Serv Saude.

[2] Seely JM, Alhassan T (2018). Screening for breast cancer in 2018-what should we be doing today?. Curr Oncol.

[3] Lauby-Secretan B, Scoccianti C, Loomis D, Benbrahim-Tallaa L, Bouvard V, Bianchini F, Straif K (2015). Breast-cancer screening--viewpoint of the IARC Working Group. N Engl J Med.

[4] Broeders M, Moss S, Nyström L (2012). The impact of mammographic screening on breast cancer mortality in Europe: a review of observational studies. J Med Screen.

[5] Duffy SW, Vulkan D, Cuckle H (2020). Effect of mammographic screening from age 40 years on breast cancer mortality (UK Age trial): final results of a randomised, controlled trial. Lancet Oncol.

[6] da Costa Vieira RA, Biller G, Uemura G, Ruiz CA, Curado MP (2017). Breast cancer screening in developing countries. Clinics (Sao Paulo).

[7] Magny SJ, Shikhman R, Keppke AL (2024). Breast imaging reporting and data system. StatPearls [Internet].

[8] Sickles EA, D’Orsi CJ, Bassett LW (2013). ACR BI-RADS mammography. ACR BI-RADS atlas, breast imaging reporting and data system, 5th edition.

[9] Kolak A, Kamińska M, Sygit K, Budny A, Surdyka D, Kukiełka-Budny B, Burdan F (2017). Primary and secondary prevention of breast cancer. Ann Agric Environ Med.

[10] Roy M, Fowler AM, Ulaner GA, Mahajan A (2023). Molecular classification of breast cancer. PET Clin.

[11] Ryu EB, Chang JM, Seo M, Kim SA, Lim JH, Moon WK (2014). Tumour volume doubling time of molecular breast cancer subtypes assessed by serial breast ultrasound. Eur Radiol.

[12] Pisano ED, Gatsonis C, Hendrick E (2005). Diagnostic performance of digital versus film mammography for breast-cancer screening. N Engl J Med.

[13] Sarmiento DD, Tumas N, Pereyra SA, Scruzzi GF, Pou SA (2024). Social determinants of breast cancer screening: a multilevel analysis of proximal and distal factors related to the practice of mammography. Cancer Epidemiol Biomarkers Prev.

[14] Mihai AM, Ianculescu L, Cretoiu D, Suciu N (2024). Breast cancer screening in Romania: challenges and opportunities for early detection. Acta Endocrinol (Buchar).

[15] (2024). Criteria for a Population Screening Program. https://www.gov.uk/government/publications/evidence-review-criteria-national-screening-programmes/criteria-for-appraising-the-viability-effectiveness-and-appropriateness-of-a-screening-programme.

[16] (2023). Supplemental screening as an adjunct to mammography for breast cancer screening in people with dense breasts: a health technology assessment. Ont Health Technol Assess Ser.

[17] Sprague BL, Coley RY, Lowry KP (2023). Digital breast tomosynthesis versus digital mammography screening performance on successive screening rounds from the Breast Cancer Surveillance Consortium. Radiology.

[18] Miglioretti DL, Abraham L, Sprague BL (2024). Association between false-positive results and return to screening mammography in the Breast Cancer Surveillance Consortium cohort. Ann Intern Med.

[19] Bernstein MH, Baird GL, Lourenco AP (2022). Digital breast tomosynthesis and digital mammography recall and false-positive rates by time of day and reader experience. Radiology.

